# 1348. Identification of GB-0669, a Novel S-2 Targeted Spike Monoclonal Antibody in Development for SARS-CoV-2 Prophylaxis

**DOI:** 10.1093/ofid/ofad500.1185

**Published:** 2023-11-27

**Authors:** Francesco Borriello, Grace Marden, Hongru Li, Eric Carlin, Geetika Sharma, Monica Menzenski, Anne Jecrois, Eugene Palovcak, Dana Lord, Jenhan Tao, Gevorg Grigoryan, Adam Root, Kristen Hopson, Daria Hazuda, Heather A Van Epps, Alex Ramos, Nathan H Joh, Pamela Russell, Joseph D Garlick

**Affiliations:** Generate Biomedicines, Lexington, Massachusetts; Generate Biomedicines, Lexington, Massachusetts; Generate Biomedicines, Lexington, Massachusetts; Generate Biomedicines, Lexington, Massachusetts; Generate Biomedicines, Lexington, Massachusetts; Generate Biomedicines, Lexington, Massachusetts; Generate Biomedicines, Lexington, Massachusetts; Generate Biomedicines, Lexington, Massachusetts; Generate Biomedicines, Lexington, Massachusetts; Generate Biomedicines, Lexington, Massachusetts; Generate Biomedicines, Lexington, Massachusetts; Generate Biomedicines, Lexington, Massachusetts; Generate Biomedicines, Lexington, Massachusetts; Generate Biomedicines, Lexington, Massachusetts; Generate Biomedicines, Lexington, Massachusetts; Generate Biomedicines, Lexington, Massachusetts; Generate Biomedicines, Lexington, Massachusetts; Generate Biomedicines, Lexington, Massachusetts; Generate:Biomedicines, Andover, Massachusetts

## Abstract

**Background:**

The clinical benefit of anti-spike mAbs for prophylaxis and treatment of SARS-CoV-2 is now well established (Baum 2020, Loo 2022, Westendorf 2022, Jones 2021, Kreuzberger 2021, Cathcart 2021, Hirsch 2022, Shi 2020); However, the evolution of SARS CoV-2 due to immune pressure has compromised the utility of all previously approved mAbs which target the variable, immune dominant receptor binding domain of the Spike protein. Despite the success of vaccination protecting the general population, the immunocompromised and elderly remain at high risk of serious disease and mortality from SARS CoV-2 infection. Therefore, there is a need to develop mAbs that remain active across current and emergent SARS CoV-2 variants for such populations.

**Methods:**

The Generate Platform uses machine learning (ML) models to generate therapeutics with desirable properties. We used this platform to explore the amino acid sequence landscape compatible with the binding conformation and breadth of neutralization for mAbs targeting the conserved S-2 stem helix of Spike. While S-2 directed antibodies can broadly neutralize diverse coronaviruses, they are generally limited by potency. We employed an iterative computation- experimentation loop to design antibodies that bind to the S-2 stem helix, optimizing for potency, breadth and developability.

**Results:**

The Generate Platform was employed to identify S-2 targeted Spike antibodies with high potency and breadth of neutralization resulting in GB-0669. GB-0669 exhibits sub-nM neutralization against all SARS-CoV-2 variants and Omicron sublineages including the highly immune evasive strain XBB. GB-0669 also displays sub-nM neutralization against sarbecoviruses associated with previous epidemics (SARS-CoV-1) and those of pandemic potential (WIV1, bat SARS-like coronavirus). The GB 0669 epitope was mapped by co-crystallization and is consistent with the observed breadth; all key interactions are >99% conserved with no evidence of immune escape.

**Conclusion:**

GB-0669, a novel mAB targeting the conserved S-2 region of Spike, is hypothesized to be effective for prophylaxis of COVID-19 across all Omicron sublineages and future variants as well as CoVs with pandemic potential.

**Disclosures:**

**Francesco Borriello, MD PhD**, Generate Biomedicines: Employee **Gevorg Grigoryan, PhD**, Generate Biomedicines: Chief Technology Officer|Generate Biomedicines: Ownership Interest|Generate Biomedicines: Stocks/Bonds **Daria Hazuda, PhD**, Generate Biomedicines: Employee|Generate Biomedicines: Stocks/Bonds|Merck and Co: Employee|Merck and Co: Stocks/Bonds **Nathan H. Joh, PhD**, Generate Biomedicines: Employee at Generate Biomedicines

